# Tissue factor activity and 1-year mortality in patients with active cancer and acute ischemic stroke: findings from the SCAN study

**DOI:** 10.1016/j.rpth.2026.103347

**Published:** 2026-01-16

**Authors:** Yasufumi Gon, Tomohiro Kawano, Takaya Kitano, Hiroshi Yamagami, Soichiro Abe, Hiroyuki Hashimoto, Nobuyuki Ohara, Daisuke Takahashi, Yuko Abe, Tsutomu Takahashi, Junji Takasugi, Hideaki Kanki, Tsutomu Sasaki, Manabu Sakaguchi

**Affiliations:** 1 Department of Neurology, Graduate School of Medicine, The University of Osaka, Japan; 2 Department of Medical Innovation, Academic Clinical Research Center, The University of Osaka Hospital, Japan; 3 Department of Stroke Neurology, National Hospital Organization Osaka National Hospital, Osaka, Japan; 4 Department of Neurology, National Cerebral and Cardiovascular Center, Osaka, Japan; 5 Department of Neurology, Osaka Rosai Hospital, Osaka, Japan; 6 Department of Neurology, Kobe City Medical Center General Hospital, Hyogo, Japan; 7 Department of Neurology, National Hospital Organization Osaka Minami Medical Center, Osaka, Japan; 8 Department of Neurology, Yodogawa Christian Hospital, Osaka, Japan; 9 Department of Neurology, Hoshigaoka Medical Center, Osaka, Japan; 10 Department of Neurology, Osaka General Medical Center, Osaka, Japan

**Keywords:** cancer, ischemic stroke, mortality, prognosis, tissue factor

## Abstract

**Background:**

Tissue factor (TF), the cellular receptor for plasma factor (F)VII/FVIIa and a key initiator of the extrinsic coagulation pathway, plays a central role in cancer-associated thrombosis. However, its prognostic significance in patients with active cancer (AC) and acute ischemic stroke (AIS) remains unclear.

**Objectives:**

This study evaluates the association between plasma TF activity and outcome in patients with AC and AIS.

**Methods:**

We analyzed data from the SCAN study, a prospective, multicenter, observational study conducted in Japan. Blood samples were obtained immediately after admission, prior to any stroke treatment. TF activity was measured using a chromogenic assay. Patients were dichotomized at the median TF activity. Kaplan–Meier survival and Cox proportional hazards models were used to assess 1-year mortality, and restricted cubic spline analysis was conducted to explore nonlinear associations between TF activity and mortality.

**Results:**

Among 135 patients with AC and AIS in the SCAN study database, 84 had available TF activity data. The median TF activity was 32.0 pM (IQR, 21.1-61.6 pM). Compared with the low TF activity group, the high TF activity group had a higher prevalence of distant metastasis (69% vs 40%; *P* = .009) and elevated D-dimer levels (median, 10.9 vs 2.7 μg/mL; *P* < .001). Kaplan–Meier analysis revealed significantly higher mortality in the high TF activity group (log-rank test, *P* < .001). High TF activity remained independently associated with increased mortality after adjustment for confounders (hazard ratio, 3.03; 95% CI, 1.29-7.12; *P* = .011).

**Conclusion:**

Elevated TF activity was independently associated with 1-year mortality in patients with AC and AIS. TF activity represents a potential prognostic biomarker in this population.

## Essentials

• TF is a key initiator of coagulation and may link cancer with stroke.
• We analyzed patients with active cancer and acute ischemic stroke.
• Higher TF activity independently predicted 1-year mortality.
• TF measurement may provide prognostic value in cancer-associated stroke.

## 1 Introduction

Ischemic stroke is the most common arterial thromboembolism in patients with cancer [[1], [2], [3]] and carries an approximately 2-fold increased risk of mortality relative to ischemic stroke in the general population [[4], [5], [6]]. In patients with cancer, ischemic strokes can occur even in the absence of conventional vascular risks or identifiable embolic sources, suggesting an underlying cancer-associated coagulopathy [[7], [8], [9]]. Cancer-associated strokes are typically characterized by multiple infarctions on neuroimaging and poor clinical outcomes [[7], [8], [9], [10], [11]]. Nevertheless, prognosis can vary substantially depending on individual patient characteristics [[11], [12], [13], [14], [15]]. Several clinical and biological factors have been associated with prognosis, including initial stroke severity [16,17], diabetes mellitus [14], distant metastasis [11,14,18], venous thromboembolism [11], cryptogenic stroke mechanisms [13,15], and biomarkers such as D-dimer levels [11,13,[19], [20], [21], [22]], C-reactive protein [19], neutrophil count [23], and von Willebrand factor (VWF) [24]. Although cancer-associated strokes are increasingly recognized as a distinct clinical entity, reliable biomarkers for outcome prediction and clinical decision making remain under investigation.

Tissue factor (TF) is the cellular receptor for plasma factor (F)VII/FVIIa and triggers blood coagulation [25]. It is expressed by tumor cells and activated monocytes, and elevated circulating TF expression or activity—particularly TF-bearing microparticles—have been implicated in thrombin generation, systemic thrombosis, tumor progression, and metastasis [[25], [26], [27], [28], [29]]. These findings raise the possibility that TF may also play a role in the pathophysiology and prognosis of cancer-associated stroke. However, the clinical and prognostic significance of TF in patients with active cancer (AC) and acute ischemic stroke (AIS) remains poorly understood. Whether TF serves merely as a surrogate marker of disease burden or whether it acts as a predictor of mortality in this population remains unclear. Understanding the prognostic role of TF may help clarify its pathophysiological significance in cancer-associated stroke.

Several studies have demonstrated that TF antigen and TF-bearing microparticles are useful biomarkers for assessing thrombotic risk in patients with cancer [25,28,30]. However, measuring TF activity can provide a more direct evaluation of the functional procoagulant potential of TF, offering a physiologically relevant indicator of the hypercoagulable state. Moreover, because TF is linked to both cancer activity and coagulation activation, it may offer additive prognostic information beyond established biomarkers such as D-dimer.

We have conducted a multicenter, prospective observational study to investigate the prognosis of patients with AC and AIS in Japan (Ischemic Stroke Patients with Cancer and Neoplasia [SCAN] study) [11,24]. In the SCAN study, blood samples were collected immediately after admission before any stroke treatment initiation, allowing for the evaluation of coagulation-related biomarkers in the hyperacute phase. We previously reported that elevated VWF antigen levels were associated with poor outcomes in this population [24].

The measurement of TF activity may more directly reflect the physiological procoagulant potential than assessments of TF antigen or TF-bearing microparticles. In this study, we investigated the association between plasma TF activity and prognosis in patients with AC and AIS. We hypothesized that elevated plasma TF activity is associated with outcomes in this population.

## 2 Methods

### 2.1 Ethical approval

This study was conducted in accordance with the Declaration of Helsinki for investigations involving humans, and the study protocol was approved by the institutional review board of the University of Osaka Hospital (approval number: 15346-10).

### 2.2 Participants

We used data from the SCAN study, a prospective, multicenter, observational study conducted in Japan. Details are described elsewhere [11,24]. Briefly, patients with AIS and malignancies were enrolled in the study and followed up until 1 year after stroke. In the current study, similar to previous reports [11,24], we identified patients in the SCAN database who met the following criteria: (1) AIS within 14 days following symptom onset, (2) age of ≥20 years, and (3) presence of AC at the time of stroke onset. Among these patients, we included those for whom TF data were available. AC was defined as a diagnosis of cancer, either treated in the last 6 months before admission or untreated or the presence of metastatic disease. Patients with a history of cancer but who did not meet the definition of active disease were not included in this study.

### 2.3 Variables

We extracted the following variables from the SCAN database: age, sex, hypertension, dyslipidemia, diabetes mellitus, smoking, atrial fibrillation, past stroke, antithrombotic (antiplatelet and/or anticoagulant) medication before stroke onset, stroke subtype, National Institute of Health Stroke Scale (NIHSS), prestroke modified Rankin Scale, infarcts pattern of brain imaging, use of recombinant-tissue plasminogen activator, mechanical thrombectomy, stroke recurrence, and major bleeding. Stroke recurrence was defined as a new neurologic deficit together with corresponding evidence of acute ischemia or hematoma on brain imaging (computed tomography and/or magnetic resonance imaging). Blood samples were collected immediately after admission, and plasma D-dimer, high-sensitivity C-reactive protein (hsCRP) levels, VWF antigen levels [24], and TF activity were quantified. Cancer-related data including cancer type, distant metastasis, adenocarcinoma, and cancer treatment (cancer surgery, chemotherapy, and radiotherapy) were also obtained.

### 2.4 Measurement of TF activity

Blood samples were obtained immediately after admission before any stroke treatment was initiated and stored at –80 °C until analysis. Platelet-poor plasma was prepared by centrifuging whole blood at 1500 × *g* for 15 minutes at room temperature (18-25 °C). The plasma supernatant was subsequently collected from at least 5 mm above the buffy coat to minimize platelet contamination. TF activity was measured using a chromogenic assay kit (Human TF Chromogenic Activity Assay Kit; Assaypro; Catalog No. CT1002b), according to the manufacturer’s instructions (https://assaypro.com/wp-content/uploads/2025/08/CT1002b.pdf). Briefly, the assay quantifies TF/FVIIa-dependent activation of FX to FXa, which cleaves a chromogenic substrate, and the absorbance at 405 nm is measured to determine TF activity based on a standard curve. All samples were assayed in duplicate to ensure reproducibility.

### 2.5 Outcomes

The primary outcome was all-cause mortality within 1 year from stroke onset. Follow-up data were collected from the SCAN databases.

### 2.6 Statistical analysis

Descriptive statistics were presented as the median with IQR for continuous variables and as frequencies with percentages for categorical variables. First, the distribution of TF activity was illustrated. To investigate the relationship between coagulation abnormalities and inflammatory response, correlations between TF activity and levels of D-dimer and hsCRP were examined using Spearman rank correlation coefficients. TF activity was compared according to adenocarcinoma and distant metastasis status using the Mann–Whitney U-test. Next, patients were categorized into 2 groups based on the median TF activity, and clinical characteristics were compared between the groups. Subsequently, survival differences were assessed using Kaplan–Meier curves. Finally, we evaluated the association of TF activity and prognosis. TF activity was dichotomized at the median value, and Cox proportional hazards regression analysis was performed to estimate hazard ratios (HRs) and corresponding 95% CIs. The following multivariable models were constructed based on previous reports: model 1: age and sex; model 2: model 1 + distant metastasis + D-dimer; model 3: model 2 + hsCRP; model 4: model 3 + VWF; and model 5: model 4 + NIHSS, prestroke antithrombotic therapy, and stroke subtype. In addition, restricted cubic spline analysis was performed to visualize the dose–response relationship between TF activity and 1-year mortality risk. A Cox proportional hazards model with 4 knots was fitted with log (TF activity) as the primary predictor, adjusted for age, sex, distant metastasis, D-dimer, hsCRP, VWF, NIHSS, prestroke antithrombotic therapy, and stroke subtype. The predicted HRs with 95% CIs were calculated using the Predict function in the R software (R Foundation for Statistical Computing), with the median log (TF activity) as the reference value (HR, 1). The dose–response curve and 95% confidence band were visualized using ggplot2 in the R software.

All statistical analyses were performed using the R software, version 4.4.3. A 2-sided *P* value of <.05 was considered statistically significant.

## 3 Results

The SCAN study database included 135 patients with AC and AIS. TF activity data were available for 84 of these patients. A comparison of patient characteristics between the included and excluded cohorts is shown in Supplemental Table S1.

### 3.1 Distribution of TF activity

**Figure 1:**
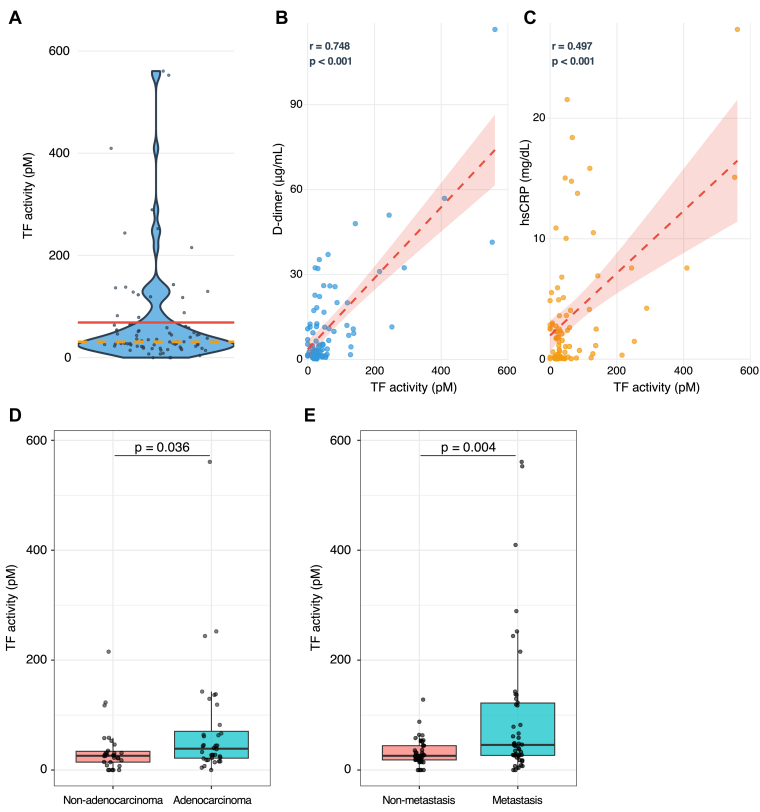
Tissue factor (TF) activity distribution and correlation with D-dimer and high-sensitivity C-reactive protein (hsCRP) levels. (A) Distribution of TF activity. The red line indicates the mean value (68.3 pM), and the orange dashed line indicates the median value (32.0 pM). (B) Association between TF activity (x-axis) and D-dimer levels (y-axis). A significant positive correlation was observed between TF activity and D-dimer levels (Spearman *r* = 0.748; *P* < .001). (C) Association between TF activity (x-axis) and hsCRP levels (y-axis). A significant positive correlation was observed between TF activity and hsCRP levels (Spearman *r* = 0.497; *P* < .001). (D) TF activity was significantly higher in patients with adenocarcinoma than that in those without (*P* = .036). (E) Similarly, TF activity was also significantly elevated in patients with distant metastasis (*P* = .004). Adenocarcinoma information was missing in 18 cases (14 from the high TF activity group and 4 from the low TF activity group), leaving 66 cases for analysis.

Figure 1 shows the distribution of TF activity, correlation of TF activity and levels of D-dimer and hsCRP, and comparison of TF activity with or without adenocarcinoma and distant metastasis. A significant correlation was observed between TF activity and D-dimer levels (*r* = 0.748; *P* < .001), as well as between TF activity and hsCRP levels (*r* = 0.497; *P* < .001). TF activity was higher in cases with adenocarcinoma and distant metastasis than those in without. TF activity did not differ by cancer treatment status (Supplemental Table S2).

### 3.2 Clinical characteristics according to TF activity

Table 1 presents the characteristics of the cohort based on the TF activity. The median TF activity was 32.0 pM (IQR, 21.1-61.6 pM). Compared with the low TF activity group, the high TF activity group had a higher prevalence of distant metastasis (69% vs 40%; *P* = .009) and elevated D-dimer levels (median, 10.9 vs 2.7 μg/mL; *P* < .001). The high TF activity group also had higher hsCRP (median, 2.50 vs 1.29 mg/dL; *P* = .013) and VWF (median, 266% vs 210%; *P* = .042) levels.

**Table 1:** Clinical characteristics based on the TF activity.

Characteristic	Low TF activity group (0-32.0 pM), *n* = 42	High TF activity group (32.0-560.8 pM), *n* = 42	*P*
Age (y)	75 (69-81)	77 (73-83)	.25
Female	38 (16)	40 (17)	.82
Hypertension	60 (25)	55 (23)	.66
Dyslipidemia	36 (15)	36 (15)	>.99
Diabetes mellitus	21 (9)	29 (12)	.45
Atrial fibrillation	26 (11)	21 (9)	.61
Smoking	21 (9)	17 (7)	.58
Past stroke	33 (14)	10 (4)	.008
Prestroke antithrombotic medication	45 (19)	29 (12)	.11
Stroke subtypes			.03
SVO	7 (3)	2 (1)	
LAA	17 (7)	7 (3)	
CES	21 (9)	19 (8)	
Others	12 (5)	0 (0)	
Cryptogenic	43 (18)	71 (30)	
NIHSS	3.5 (1.0-8.8)	5.0 (2.2-12.8)	.19
Prestroke mRS ≤ 2	81 (34)	60 (24)	.03
Multiple infarcts	33 (14)	61 (25)	.01
DVT/PE complication	5 (2)	12 (5)	.24
rtPA	7 (3)	5 (2)	.65
Endovascular therapy	14 (6)	12 (5)	.75
Distant metastasis	40 (17)	69 (29)	.009
Adenocarcinoma	42 (16)	71 (20)	.018
Cancer treatment	81 (34)	68 (28)	.18
Cancer surgery	26 (11)	17 (7)	.31
Chemotherapy	52 (22)	56 (23)	.73
Radiotherapy	12 (5)	7 (3)	.48
Stroke recurrence	7 (3)	10 (4)	.69
Major bleeding	2 (1)	12 (5)	.09
Death	26 (11)	69 (29)	<.001
D-dimer (μg/mL)	2.7 (1.3-8.8)	10.9 (4.3-26.0)	<.001
hsCRP (mg/dL)	1.29 (0.17-2.55)	2.50 (0.45-7.57)	.01
VWF (%)	210 (160-310)	266 (217-317)	.04

Continuous variables are presented as median (IQR) and categorical variables as % (*n*). All participants in this study were Japanese (Asian).

CES, cardioembolism; DVT, deep venous thrombosis; hsCRP, high-sensitivity C-reactive protein; LAA, large artery atherosclerosis; mRS, modified Rankin Scale; NIHSS, National Institute of Health Stroke Scale; PE, pulmonary embolism; rtPA, recombinant tissue plasminogen activator; SVO, small vessel occlusion; TF, tissue factor; VWF, von Willebrand factor.

**Figure 2:**
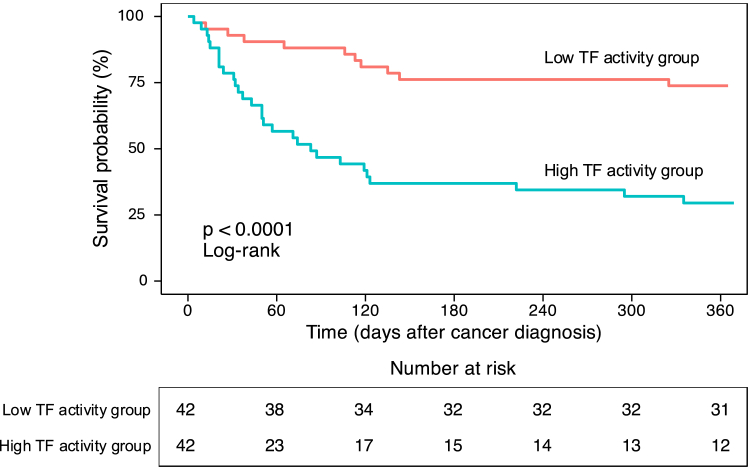
Kaplan–Meier survival curves by tissue factor (TF) activity. One-year survival was significantly lower in patients with high TF activity than that in those with low TF activity (log-rank test, *P* < .001).

Figure 2 illustrates the Kaplan–Meier survival curves stratified by TF activity. Patients in the high TF activity group had significantly poorer survival than those in the low TF activity group during the 1-year follow-up period (log-rank test, *P* < .001).

### 3.3 Association between TF activity and mortality

**Figure 3:**
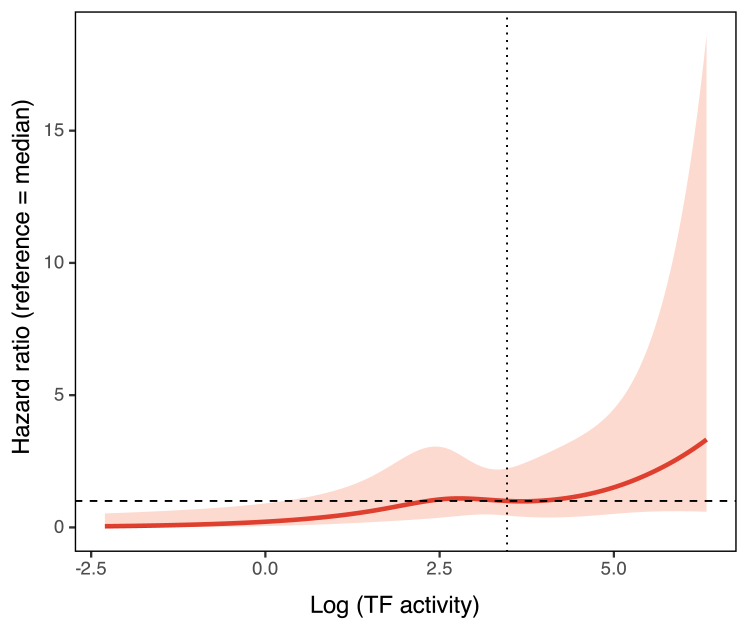
Association between tissue factor (TF) activity and 1-year mortality based on restricted cubic spline analysis. Adjusted hazard ratios (solid red line) and 95% CIs (shaded area) for 1-year mortality according to TF activity modeled using restricted cubic splines. The reference value is the median TF activity (logTF, 3.5), corresponding to a hazard ratio of 1.0. Hazard ratios were adjusted for age, sex, distant metastasis, D-dimer, high-sensitivity C-reactive protein, von Willebrand factor, National Institute of Health Stroke Scale, prestroke antithrombotic therapy, and stroke subtype.

Table 2 summarizes the HRs for 1-year mortality according to TF activity dichotomized at the median value. In the univariable analysis, high TF activity was significantly associated with increased mortality (HR, 4.04; 95% CI, 2.00-8.13; *P* < .001). This association remained significant after adjusting for potential confounders across multiple models (models 1-5). The fully adjusted model 5 yielded an HR of 3.03 (95% CI, 1.29-7.12; *P* = .011). Figure 3 shows the results of restricted cubic spline analysis. The reference value was set at the median TF activity (32.0 pM; HR, 1). The restricted cubic spline analysis demonstrated a gradual increase in the risk of 1-year mortality with higher TF activity, after adjustment for potential confounders (model 5). The association between log-transformed TF activity and mortality was statistically significant (*P* = .015), and the relationship appeared approximately linear (*P*_nonlinearity_ = .55).

**Table 2:** HRs for 1-year mortality by tissue factor activity in Cox regression models.

Model		HR (95% CI)	*P*
Univariable		4.04 (2.00-8.13)	<.001
Multivariable	Model 1	4.12 (2.04-8.32)	<.001
	Model 2	3.69 (1.71-7.95)	<.001
	Model 3	3.64 (1.68-7.89)	.001
	Model 4	3.49 (1.57-7.78)	.002
	Model 5	3.03 (1.29-7.12)	.011

Model 1: age and sex; model 2: age, sex, distant metastasis, and D-dimer; model 3: age, sex, distant metastasis, D-dimer, and high-sensitivity C-reactive protein; model 4: age, sex, distant metastasis, D-dimer, high-sensitivity C-reactive protein, and von Willebrand factor; and model 5: age, sex, distant metastasis, D-dimer, high-sensitivity C-reactive protein, von Willebrand factor, National Institute of Health Stroke Scale, prestroke antithrombotic therapy, and stroke subtype.

HR, hazard ratio.

## 4 Discussion

Cancer-associated ischemic stroke is often linked to advanced-stage malignancy [[8], [9], [10], [11],^13^]. Therefore, prognostic biomarkers are clinically important for guiding both cancer treatment and stroke management after stroke onset [8,29]. In the current study, we used the SCAN database and found that patients in the high TF activity group had poorer 1-year survival than those in the low TF activity group. Elevated TF activity was independently associated with increased risk of mortality. These findings underscore the importance of TF activity in cancer-associated ischemic stroke and contribute to the advancement of research in this field.

It is noteworthy that TF activity was independently associated with poor prognosis, even after adjustment for known prognostic factors. Previous studies have focused on coagulation-related biomarkers such as D-dimer and thrombin–antithrombin complex as indicators of hypercoagulability in patients with cancer-associated stroke [9,11,13,19,22]. Additionally, markers related to platelet activation and thrombosis, such as P-selectin and VWF, have also received attention [22,24]. However, to our knowledge, no previous studies have specifically examined plasma TF activity in patients with AC and AIS. TF plays a central role in the initiation of the extrinsic coagulation pathway and is upregulated in various malignancies, where it contributes to tumor progression and thrombosis [28,31,32]. Therefore, in patients with both cancer and ischemic stroke, TF activity may reflect the intersection of tumor biology and coagulation dysregulation and thereby explain its association with poor clinical outcomes.

The significance of measuring TF activity in patients with cancer-associated stroke remains unclear. D-dimer, hsCRP, and VWF reflect hypercoagulability, systemic inflammation, and endothelial dysfunction, respectively. In contrast, TF activity represents the upstream trigger of the coagulation cascade and is mechanistically linked to both cancer activity and activation coagulation [25]. Therefore, we thought that measurement of TF activity may provide additive prognostic information beyond these established biomarkers by capturing the primary procoagulant drive in cancer-associated stroke.

Elevated TF activity was frequently observed in patients with adenocarcinoma and distant metastasis, suggesting a close relationship between tumor biology and coagulation dysfunction. TF is known to be overexpressed in various adenocarcinomas, including pancreatic [33,34] and gastric cancers [35], where elevated expression is associated with tumor progression. Another study has shown that TF activity was significantly higher in adenocarcinoma than that in nonadenocarcinoma among cancer patients [36]. Additionally, TF-bearing microparticles have been identified as important mediators linking cancer to thrombosis [[37], [38], [39], [40]], and TF expression has been associated with metastatic potential in lung adenocarcinoma [41]. Taken together, our results highlight the potential utility of TF as a circulating biomarker that reflects both cancer aggressiveness and thrombogenicity and as a promising target for risk stratification in cancer-related stroke.

The high prevalence of cryptogenic stroke in the high TF activity group (71% vs 43%) supports the hypothesis that elevated TF activity may reflect cancer-related hypercoagulability in patients without other identifiable stroke etiologies, reinforcing the role of TF as a biomarker for cancer-associated stroke mechanisms. The association between high TF activity and multiple infarcts (61% vs 33%; *P* = 0.01) is consistent with the systemic hypercoagulable state characteristic of cancer-associated stroke, where widespread thrombotic events may occur simultaneously.

Several methodological considerations should be noted regarding the measurement of TF activity in plasma [42]. First, plasma contains endogenous FVII/FVIIa and FX that may interfere with the results. Second, plasma contains TF pathway inhibitor that may interfere with the TF–FVIIa complex formation. Finally, no anti-TF antibody is used to distinguish between TF-dependent and TF-independent FXa generation. Despite these limitations, we believe that TF activity in plasma provides an integrated estimate of the functional procoagulant potential, which may have pathophysiological and prognostic relevance in patients with cancer-associated stroke. In addition, the standard measurement of TF activity in humans is not established yet. Future studies are needed.

A major strength of this study is the use of a prospective, multicenter SCAN database [11,24]. However, several limitations should be acknowledged. First, stroke recurrence was observed in only 7 of the 84 patients included in the analysis—4 in the high TF activity group and 3 in the low TF activity group—rendering meaningful statistical analysis of the association between TF activity and recurrence unfeasible. Second, since our data were limited to the patients with AC and AIS, we could not clearly distinguish whether elevated TF activity was attributable to cancer, stroke, or a combination of both. Third, TF activity was analyzable in only 84 of the 135 patients included in the registry, which may have introduced selection bias. Fourth, because all participants were Japanese (Asian), racial and ethnic diversity was not represented in this study. Therefore, the generalizability of our findings to other ethnic populations may be limited. Finally, this study was exploratory in nature, and the findings should be interpreted as hypothesis generating. Although TF activity showed a significant association with 1-year mortality, our results require validation in larger, independent, and more diverse patient cohorts before any clinical application can be considered. Therefore, the potential use of TF activity as a prognostic biomarker remains preliminary.

In conclusion, elevated TF activity was independently associated with increased 1-year mortality in patients with AC and AIS. Our findings highlight the potential value of TF as a prognostic biomarker and suggest that it may reflect an underlying coagulation status. Further studies with larger cohorts and longitudinal biomarker assessments are warranted to validate these findings.
