# Supplementary material for: Tissue factor activity and 1-year mortality in patients with active cancer and acute ischemic stroke: findings from the SCAN study
Source: Res Pract Thromb Haemost. 2026 Jan 16;10(1):103347. doi: 10.1016/j.rpth.2026.103347 (PMC12906000; doi:10.1016/j.rpth.2026.103347)
Supplement: Supplemental Tables S1 and S2 [file mmc1.docx]

**Supplemental Material**

**Table S1. Clinical characteristics: included cohort vs excluded cohort**

**Table S2. Clinical characteristics according to cancer treatment**

**Table S1. Clinical characteristics: included cohort vs excluded cohort**

|  | | Included (n=84) | Excluded (n=51) | *P* value |
| --- | --- | --- | --- | --- |
| Age | | 76 (70–81) | 74 (68–79) | 0.15 |
| Female | | 39% (33) | 37% (19) | 0.81 |
| Hypertension | | 57% (48) | 69% (35) | 0.18 |
| Hyperlipidemia | | 36% (30) | 31% (16) | 0.61 |
| Diabetes mellitus | | 25% (21) | 18% (9) | 0.32 |
| Atrial fibrillation | | 20% (24) | 18% (9) | 0.40 |
| Smoking | | 19% (16) | 18% (9) | 0.84 |
| Past stroke | | 21% (18) | 14% (7) | 0.26 |
| Pre-stroke antithrombotic medication | | 37% (31) | 20% (10) | 0.034 |
| Stroke subtypes | |  |  | 0.086 |
|  | SVO | 5% (4) | 14% (7) |  |
|  | LAA | 12% (10) | 20% (10) |  |
|  | CES | 20% (17) | 22% (11) |  |
|  | Others | 6% (5) | 10% (5) |  |
|  | Cryptogenic | 57% (48) | 35% (18) |  |
| NIHSS | | 4.0 (2.0-10.0) | 4.0 (2.0-8.5) | 0.79 |
| Pre-stroke mRS ≤2 | | 70% (59) | 84% (43) | 0.065 |
| Multiple infarcts | | 47% (39) | 43% (22) | 0.66 |
| DVT/PE complication | | 8% (7) | 6% (3) | 0.60 |
| Rt-PA | | 6% (5) | 8% (4) | 0.67 |
| Endovascular therapy | | 13% (11) | 6% (3) | 0.18 |
| Distant metastasis | | 55% (46) | 45% (23) | 0.28 |
| Adenocarcinoma | | 55% (36) | 58% (19) | 0.78 |
| Cancer treatment | | 75% (62) | 80% (41) | 0.45 |
|  | Cancer surgery | 22% (18) | 39% (20) | 0.029 |
|  | Chemotherapy | 54% (45) | 37% (19) | 0.056 |
|  | Radiotherapy | 10% (8) | 14% (7) | 0.47 |
| Stroke recurrence | | 8% (7) | 12% (6) | 0.51 |
| Major bleeding | | 7% (6) | 8% (4) | 0.88 |
| Death | | 48% (40) | 43% (22) | 0.61 |
| D-dimer, μg/mL | | 2.1 (1.2-8.6) | 5.2 (1.8–15.7) | 0.029 |
| hsCRP, mg/dL | | 1.75 (0.20–4.18) | 0.44 (0.16–3.02) | 0.12 |
| vWF, % | | 211 (99–246) | 250 (183–312) | 0.10 |

SVO, small vessel occlusion; LAA, large artery atherosclerosis; CES, cardioembolism; NIHSS, National Institute of Health Stroke Scale; mRS, modified Rankin Scale; DVT, deep venous thrombosis; PE, pulmonary embolism; Rt-PA, recombinant tissue plasminogen activator; hsCRP, high sensitivity C-reactive protein; vWF, von Willebrand factor.

**Table S2. Clinical characteristics according to cancer treatment**

|  | | Receiving cancer treatment (n=62) | Not receiving cancer treatment (n=21) | *P* value |
| --- | --- | --- | --- | --- |
| Age | | 76 (70–81) | 77 (73–84) | 0.22 |
| Female | | 31% (19) | 62% (13) | 0.011 |
| Hypertension | | 61% (38) | 48% (10) | 0.27 |
| Hyperlipidemia | | 32% (20) | 48% (10) | 0.20 |
| Diabetes mellitus | | 24% (15) | 29% (6) | 0.69 |
| Atrial fibrillation | | 26% (16) | 19% (4) | 0.53 |
| Smoking | | 19% (12) | 14% (3) | 0.60 |
| Past stroke | | 23% (14) | 19% (4) | 0.73 |
| Pre-stroke antithrombotic medication | | 39% (24) | 33% (7) | 0.66 |
| Stroke subtypes | |  |  | 0.21 |
|  | SVO | 3% (2) | 10% (2) |  |
|  | LAA | 13% (8) | 10% (2) |  |
|  | CES | 24% (15) | 10% (2) |  |
|  | Others | 8% (5) | 0% (0) |  |
|  | Cryptogenic | 52% (32) | 71% (15) |  |
| NIHSS | | 4.0 (2.0-8.8) | 7.0 (1.0-17.0) | 0.27 |
| Pre-stroke mRS ≤2 | | 73% (45) | 62% (13) | 0.36 |
| Multiple infarcts | | 45% (28) | 52% (11) | 0.57 |
| DVT/PE complication | | 10% (6) | 5% (1) | 0.48 |
| Rt-PA | | 2% (1) | 19% (4) | 0.004 |
| Endovascular therapy | | 16% (10) | 5% (1) | 0.18 |
| Distant metastasis | | 55% (34) | 57% (12) | 0.85 |
| Adenocarcinoma | | 56% (32) | 44% (4) | 0.51 |
| Stroke recurrence | | 5% (3) | 19% (4) | 0.043 |
| Major bleeding | | 6% (4) | 10% (2) | 0.64 |
| Death | | 45% (28) | 57% (12) | 0.34 |
| D-dimer, μg/mL | | 3.8 (1.6-10.9) | 14.4 (4.7–24.8) | 0.006 |
| hsCRP, mg/dL | | 1.66 (0.18–3.94) | 1.75 (0.44–5.93) | 0.52 |
| vWF, % | | 235 (179–308) | 303 (216–343) | 0.078 |
| Tissue factor, pM | | 30 (19–60) | 37 (22–79) | 0.43 |

Cancer treatment history was unknown for one of the 84 patients.

SVO, small vessel occlusion; LAA, large artery atherosclerosis; CES, cardioembolism; NIHSS, National Institute of Health Stroke Scale; mRS, modified Rankin Scale; DVT, deep venous thrombosis; PE, pulmonary embolism; Rt-PA, recombinant tissue plasminogen activator; hsCRP, high sensitivity C-reactive protein; vWF, von Willebrand factor.
